# Celastrol Induces Necroptosis and Ameliorates Inflammation via Targeting Biglycan in Human Gastric Carcinoma

**DOI:** 10.3390/ijms20225716

**Published:** 2019-11-14

**Authors:** Dandan Guo, Wei Zhang, Haijie Yang, Jiajia Bi, Yunfei Xie, Binfeng Cheng, Yan Wang, Sujuan Chen

**Affiliations:** 1 Synthetic Biology Engineering Lab of Henan Province, School of Sciences and Techanology, Xinxiang Medical University, Henan 453003, China; 52181300030@stu.ecnu.edu.cn (D.G.); 141005@xxmu.edu.cn (J.B.); 111015@xxmu.edu.cn (Y.W.); 2 College of Animal Science and Veterinary Medicine, Henan Institute of Science and Technology, Xinxiang, Henan 453003, China; xuyanzhao@hist.edu.cn; 3 School of Sciences and Technology, Xinxiang Medical University, Henan 453003, China; 131035@xxmu.edu.cn (H.Y.); xieyf@xxmu.edu.cn (Y.X.); chengbinfeng@xxmu.edu.cn (B.C.)

**Keywords:** celastrol, necroptosis, biglycan, cytokines, gastric cancer cells

## Abstract

Celastrol, a triterpene isolated from the root of traditional Chinese medicine *Thunder of God Vine*, possesses anti-cancer and anti-inflammatory activity to treat rheumatoid disease or as health product. Necroptosis is considered as a new approach to overcome chemotherapeutics resistance. However, whether celastrol exerts necroptosis leading to gastric cancer cell death is still unclear. Here, for the first time we showed that celastrol induced necroptosis in HGC27 and AGS gastric cancer cell lines. More importantly, celastrol down-regulated biglycan (BGN) protein, which is critical for gastric cancer migration and invasion. Furthermore, celastrol activated receptor-interacting protein 1 and 3 (RIP1 and RIP3) and subsequently promoted the translation of mixed-lineage kinase domain-like (MLKL) from cytoplasm to plasma membrane, leading to necroptosis of gastric cancer cell, which was blocked by over-expression BGN. In addition, celastrol suppressed the release of pro-inflammatory cytokines TNF-α and IL-8 in HGC27 and AGS cells, which was reversed by over-expression BGN. Taken together, we identified celastrol as a necroptosis inducer, activated RIP1/RIP3/MLKL pathway and suppressed the level of pro-inflammatory cytokines by down-regulating BGN in HGC-27 and AGS cells, which supported the feasibility of celastrol in gastric cancer therapy.

## 1. Introduction

Gastric cancer is a common malignant tumor of digestive system worldwide, and its incidence rate is particularly high around the world, especially in eastern Asia [1,2,3]. Clinical data revealed the detection rate of early gastric cancer is very low, so most patients have reached the advanced stage because of symptoms. Chemotherapy and radiotherapy are common therapeutic strategies [4]. However, the response rate to the chemotherapeutic drugs is only between 20% and 40% [5]. Resistance to cell apoptosis is a major obstacle in gastric cancer chemotherapy. Thus, application of anticancer agents targeting non-apoptitic cell death pathways may be a novel anticancer strategy.

In recent years, it has been reported that cell death involves two kinds of mechanisms (caspase-dependent and caspase-independent). Caspase-independent cell death has been named necroptosis, which is also a kind of important programmed cell death [6]. Necrosome, a complex assembled from political signaling molecules mediates the development of necroptosis, including caspase-8, Fas-associated death domain protein (FADD), and two receptor-interacting serine/threonine-protein (RIP1 and RIP3). Many stimulis such as TNF-α induce the interaction between RIP1 and RIP3 resulting in the formation of necrosome [7,8]. After that, RIP3 is activated and in turn recruits its downstream protein mixed-lineage kinase domain-like (MLKL). Then, MLKL translocates from cytoplasm to plasma membrane, eventually forming calcium influx-mediated pore to promote necroptosis [9].

Biglycan (BGN), a proteoglycan whose core protein is glycanated by two GAG chains, is a member of the superfamily of proteoglycans called the small leucine repeat proteoglycan family (SLRP) [10]. BGN exists in almost all organs of our body, but its distribution is not uniform, which mainly distributed in the extracellular matrices and on the cell surface of some specialized cells [6,11,12,13]. The functions of BGN are dependent on the microenvironment in which it acts as a structural molecule or signaling molecule [14,15]. Therefore, it is not surprising that biglycan possesses a variety of functions involving in atherosclerotic plaques, long-term memory, bone mass and cell migration [16]. Recent studies have indicated significantly higher expression of BGN in tumor tissues compared with adjacent normal tissues, including colon tumor, ovary cancer, pancreatic adenocarcinoma, intrahepatic cholangiocarcinoma, and gastric cancer [17,18,19,20,21]. Abnormal BGN expression in tumor tissues suggests that it plays an oncogenic role in cancer migration and invasion [22,23].

There is mounting evidence that BGN not only directly triggers pro-inflammatory TLR- and inflammasomes-signaling but also stimulates the generation of pro-inflammatory cytokines (eg. TNF-α, IL-1β, IL-6 and IL-8) and ROS, which are crucial mediators of inflammation and angiogenesis in cancer development [24]. It is conceivable that the regulation of BGN in cancer is closely associated with inflammation. Large amounts of cytokines, chemokines, and ROS contributed to tumor initiation and progression. Moreover, the inflammasome as key signal in microenvironment is the hallmark in each stage of tumor development [25,26]. Despite emerging insight that BGN may affect cancer development dependent on inflammation, the relation between BGN and necroptosis in gastric cancer remains to be uncovered.

Celastrol, extracted from the root of the plant *Tripterygium wilfordii* (Thunder of God vine), is one of the quinone methide triterpenoids [27,28], which is used in traditional Chinese medicine and health products as food compound [29,30]. It is well-known that celastrol have exhibited diverse pharmacological properties for therapeutic potential, including anti-rheumatoid arthritis, antioxidant, and anti-cancer effects [31,32,33,34,35]. Recently, celastrol has also been found to inhibit gastric cancer growth by induction of apoptosis and autophagy. However, it is unknown whether necroptosis is involved in the molecular mechanism of celastrol-induced gastric cancer cell death.

In this study, for the first time, our results indicate the celastrol induced necroptosis and attenuated the release of pro-inflammatory cytokines via down-regulating BGN level in the gastric carcinoma cell lines HGC-27 and AGS cells. Furthermore, celastrol activated RIP1/RIP3/MLKL signaling pathway, leading to necroptosis.

## 2. Results

### 2.1. Celastrol Induces Gastric Cancer Cell Death, Possibly via Necroptosis

**Figure 1:**
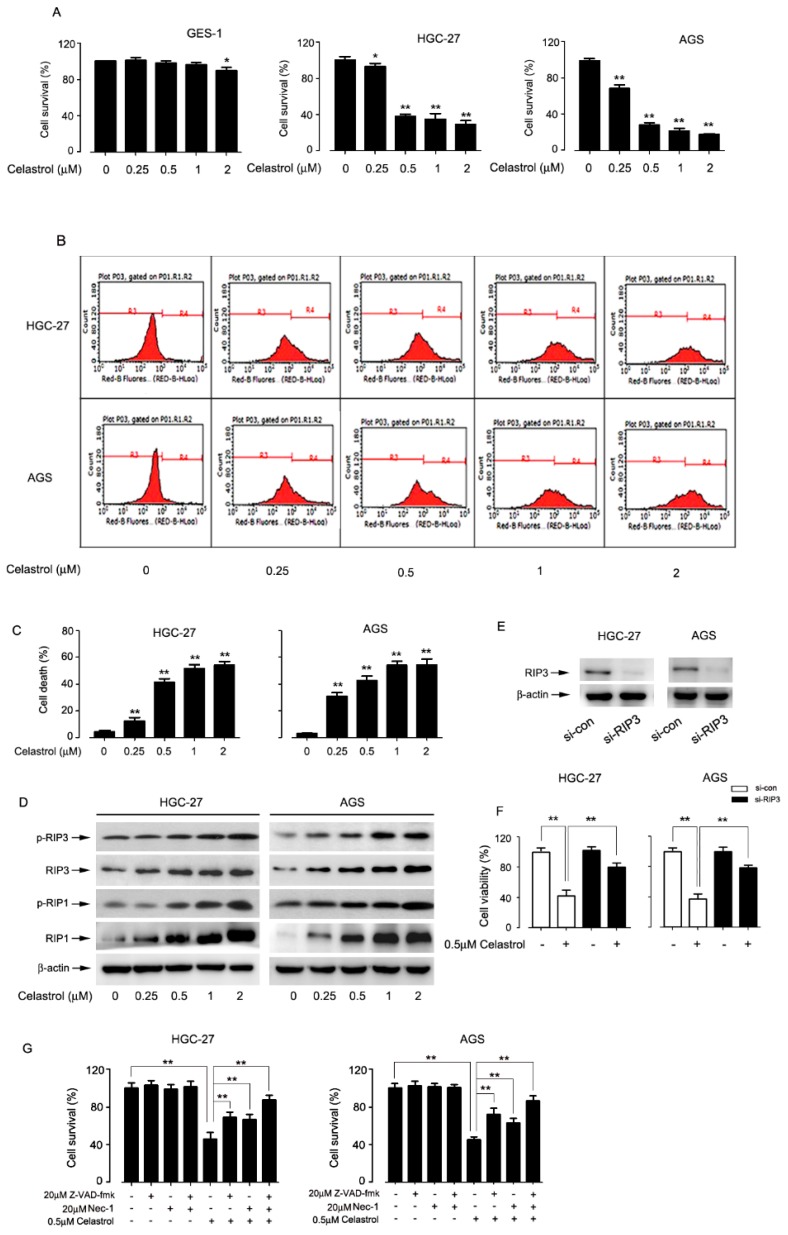
Celastrol partially induced necroptosis in gastric cancer cells. HGC-27, AGS and GES-1 cells were treated with different concentrations (0, 0.25, 0.5, 1, and 2 μM) of celastrol for 24 h. After that, (**A**) the percentage of cell survival was determined with MTT assay and (**B** and **C**) the percentage of cell death was determined with PI staining plus flow cytometry. All data were presented as the mean ± S.D. of at least three independent experiments. Significant differences compared with controls were indicated as * *P* < 0.05 and ** *P* < 0.01. (**D**) The expression of p-RIP1 and p-RIP3 were detected by Western blot analysis. β-actin was used as an internal control. (**E**) Cells were transfected with scrambled siRNA (con) or siRIP3 for 72 h prior to celastrol (0.5 μM) treatment for 24 h. The efficiency of siRIP3 in HGC-27 and AGS cells were determined by Western blot. (**F**) The MTT assay showed that RIP3 silencing significantly rescued celastrol-induced cell death in HGC-27 and AGS cells. (**G**) Cells were pretreated with/without 20 μM Z-VAD-fmk or 20 μM Nec-1 following by 0.5 μM celastrol for 24 h. After that, the percentage of cell survival was determined with MTT assay. Values were presented as mean ± SD of three determinations obtained from three different experiments, * *P* < 0.05, ** *P* < 0.01.

Recently, it was reported that natural compounds such as matrine [36], tanshinone IIA [37], shikonin [38], induced cancer cell death by necroptosis. Celastrol inhibited the growth of gastric cancer cells [39], however, whether necroptosis participated in the event of celastrol-induced cell death is unknown. To investigate whether celastrol induces necroptosis in gastric cancer cells, we first tested the cytotoxicity of celastrol in cell lines (HGC-27, AGS) and human normal gastric epithelial cell line GES-1 cells. Results from MTT assay showed that celastrol inhibited proliferation in a dose-dependent manner in two cells lines tested. However, celastrol (0–1 μM) had no effect on the survival of GES-1 cells. About 60.0% suppression rate of cell growth was produced after treatment with 0.5 μM celastrol for 24 h (Figure 1A). We further explored necroptosis by PI staining and Western blotting assay. Flow cytometry analysis indicated that celastrol induced cell death in a concentration-dependent manner (Figure 1B,C).

The expression of RIP1 and RIP3 is essential for the formation of necrosome to undergo necroptosis. Therefore, we investigated the change of RIP1 and RIP3 level in HGC-27 and AGS cells after exposed celastrol. Results from Western blotting showed that celastrol dramatically activated p-RIP1 and p-RIP3 of two cells lines tested in a dose-dependent manner (Figure 1D). To further clarify the role of necroptosis in celastrol-induced gastric cancer cell death, we used siRNA technology to interfere with RIP3 expression. As the results showed in Figure 1E,F, knockdown RIP3 significantly inhibited celastrol-induced cell death in gastric cancer cell. We also evaluated the role of apoptosis and necroptosis on celastrol-triggered cell death. From the result of Figure 1G, both apoptosis inhibitor (Z-VAD-fmk) and RIP1 inhibitor (Nec-1) in part rescued celastrol-triggered gastric cancer cell death. The combination of them had induced stronger protective effect on the cytotoxicity of celastrol in gastric cancer cell than Z-VAD-fmk + celastrol group or Nec-1 + celastrol group separately. The above data indicate that celastrol partially induces necroptosis in gastric cancer cell lines.

### 2.2. Celastrol Down-Regulated the Expression of BGN Leading to HGC-27 and AGS Cell Death

**Figure 2:**
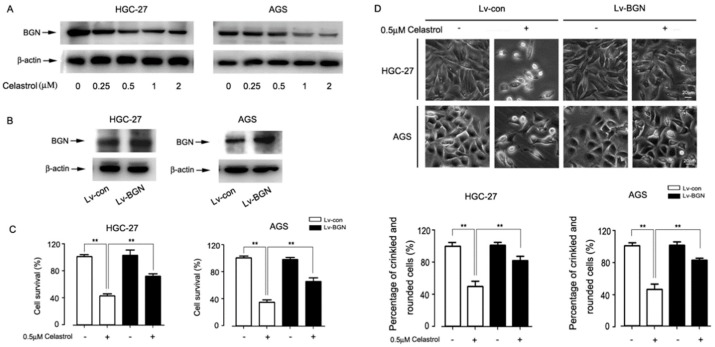
BGN down-regulated by celastrol is associated with cell death in gastric cancer cells. (**A**) Celastrol inhibited the expression of BGN in a dose dependent manne in HGC-27 and AGS cells. Cells were treated with different concentration (0, 0.25, 0.5, 1, and 2 μM) celastrol for 24 h, and BGN was detected by Western blot. (**B**) The efficiency of over-expression BGN in HGC-27 and AGS cells was determined by Western blot. (**C** and **D**) HGC-27 and AGS cells were infected by lentiviral vector for over-expression of BGN or GFP and then treated with 0.5 μM celastrol for 24 h. The percentage of cell survival was estimated by MTT assay (**C**), and the morphology of cells was assessed using an Olympus inverted phasecontrast microscope (200×) equipped with Quick Imaging system. Scale bar: 20 μm. Results are presented as mean ± SE, *n* = 6–12. * *P* < 0.05, ** *P* < 0.01 (**D**).

**Figure A1:**
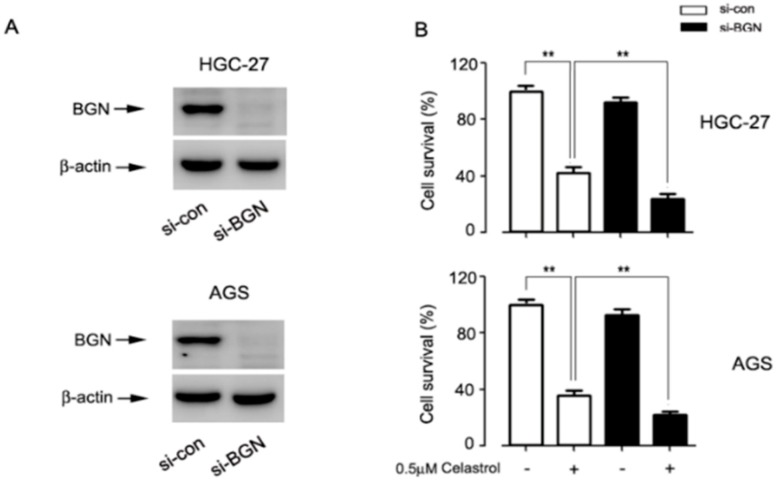
BGN silencing aggravates celastrol-induced cell death in gastric cancer cells. (**A**) Cells were transfected with scrambled siRNA (con) or siBGN for 72 h prior to celastrol (0.5 μM) treatment for24 h. The efficiency of siBGN in HGC-27 and AGS cells was determined by Western blotting. (**B**) The MTT assay showed that BGN silencing enhanced celastrol-induced cytotoxicity in HGC-27 and AGS cells. Values were presented as mean ± SD of three determinations obtained from three different experiments, * *P* < 0.05, ** *P* < 0.01.

BGN expression was upregulated in gastric cancer tissues to enhance gastric cancer invasion [6]. We therefore hypothesized that celastrol-induced cell death is closely related with BGN. The results showed that celastrol remarkably down-regulated the BGN protein level in HGC-27 and AGS cells (Figure 2A). To dissect the role of BGN in celastrol-induced cell death, endogenous BGN in HGC-27 and AGS cells was over-expressed using lentivral-mediated technology (Figure 2B). Interestingly, we found that over-expression of BGN dramatically attenuated celastrol-induced cell death (Figure 2C). In addition, by phase-contrast microscopic observation, morphological changes of HGC-27 and AGS cells, because of treatment to celastrol, included rounding and shrinkage, which was markedly abolished by over-expression of BGN (Figure 2D). To further clarify the relationship between BGN and celastrol in gastric cancer cell, BGN gene was silenced using siRNA technique. As illustrated in S1, BGN expression was remarkably knocked down in BGN-specific siRNA-transfected cells, compared with scrambled siRNA-transfected control cells (Figure A1A). MTT assay demonstrated that BGN silencing decreases the cell viability in the presence of celastrol in HGC-27 and AGS cells (Figure A1B). These results indicate that the cell death induced by celastrol in gastric cells targets BGN.

### 2.3. Celastrol Down-Regulated the Expression of BGN to Activate RIP1/RIP3 Necroptosis Signaling in HGC-27 and AGS Cells

**Figure 3:**
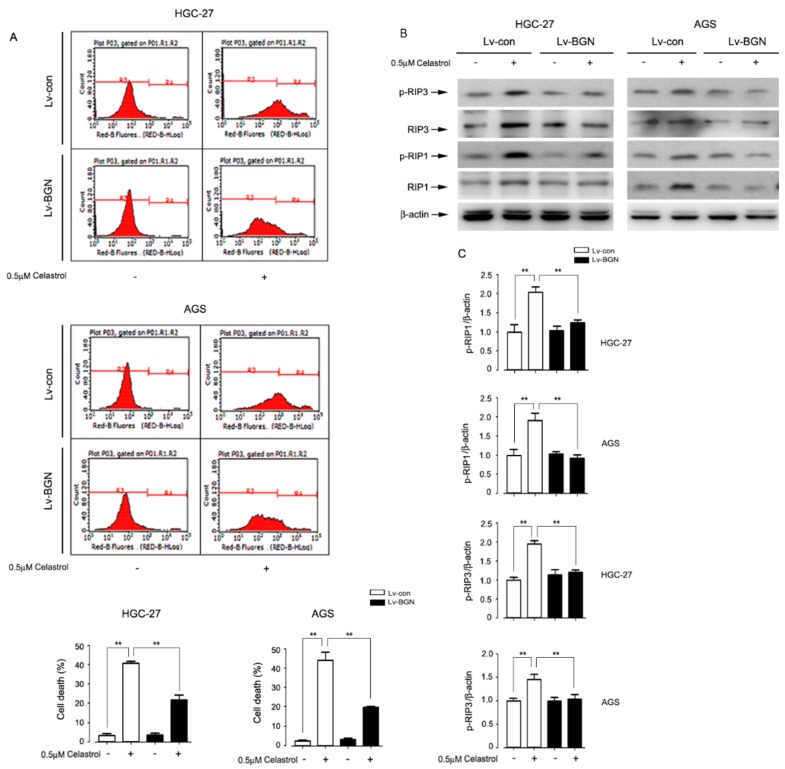
BGN down-regulated by celastrol is associated with necroptosis by regulating RIP1 and RIP3 in gastric cancer cells. BGN in HGC-27 and AGS cells was over-expressed with lentiviral vector, and then cells were treated with 0.5 μM celastrol for 24 h. The percentage of cell death was estimated by PI staining plus flow cytometry (**A**) and the expression of p-RIP1 and p-RIP3 was detected by Western blotting (**B**). The blots for p-RIP1 and p-RIP3 were semi-quantified using NIH image J (**C**). Results were presented as the mean ± S.D. of at least three independent experiments. Significant differences compared with controls were indicated as * *P* < 0.05 and ** *P* < 0.01. β-ac tin was used as an internal control.

To further test whether BGN participated in celastrol-induced necroptosis in gastric cancer, we evaluated the necroptosis by flow cytometry and Western blotting. Results showed that over-expression of BGN obviously inhibited celastrol-induced increase of necroptosis in both HGC-27 and AGS cell lines (Figure 3A). Further study with Western blotting analysis showed that BGN overexpression exhibited the effect of inhibiting celastrol-induced phosphorylation of RIP1 and RIP3 in two cells lines tested (Figure 3B,C). These data indicate that celastrol targets down-regulating BGN and then promotes RIP1/RIP3 expression, resulting in the necroptosis.

### 2.4. Celastrol Promoted the Translation of MLKL, Leading to Necroptosis in HGC-27 and AGS Cells

**Figure 4:**
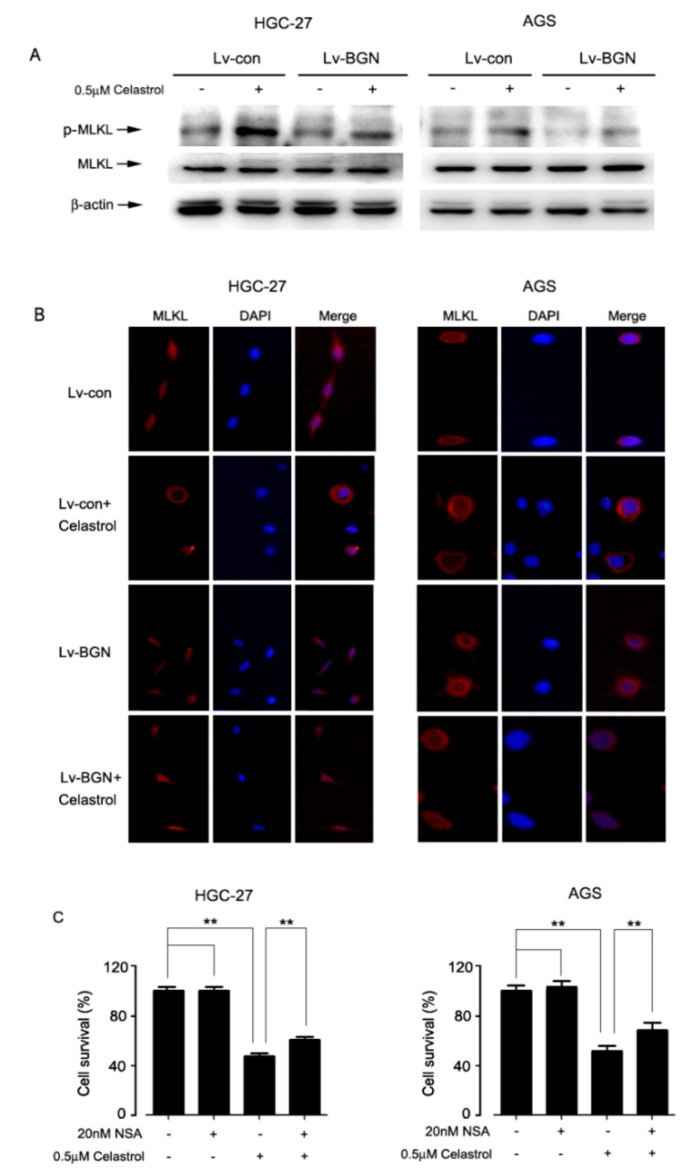
MLKL as a downstream of BGN/RIP3 is requred for celastrol inducing necroptosis. (**A**) Over-expression BGN protein blocked celastrol-induced the phosphorylation of MLKL and (**B**) the translocation from cytoplasm to plasma membrane. Subcellular localization of MLKL was analyzed by immunofluorescence and fluorescent light microscope. (**C**) Cells were pre-treated with MLKL inhibitor NSA (20 nM) for 2 h and then treated with 0.5 μM celastrol for 24 h. After then, the percentage of cell survival was estimated by MTT assay.

MLKL is the critical substrate of RIP3 in necroptosis signaling pathway. To confirm the finding that celastrol induced necroptosis, we tested whether MLKL participated in the event. As the results from Western blotting showed that celastrol induced the phosphorylation of MLKL, which was signficantly attenuated by BGN overexpression in HGC-27 and AGS cells (Figure 4A). Translocation of MLKL from cytoplasm to plasma membrane leading to fatal permeabilization of the plasma is required for necroptosis. The result from immunofluorescent staining showed that MLKL was originally located in the cytoplasm of control cells. However, most of MLKL moved to the plasma membrane after celastrol treatment, which was obviously prevented by over-expression of BGN (Figure 4B). Furthermore, the result showed that cell death caused by celastrol was significantly suppressed by MLKL-specific inhibitor necrosulphonamide (NSA) in HGC-27 and AGS cells (Figure 4C). These data together indicate that celastrol down-regulates the expression of BGN to activate RIP3/MLKL signaling, leading to necroptosis in HGC-27 and AGS cells.

### 2.5. Inhibition of Necroptosis by Nec-1 Attenuated Celastrol-Induced Cell Death in Part by Up-Regulating the Expression of BGN in HGC-27 and AGS cells

**Figure 5:**
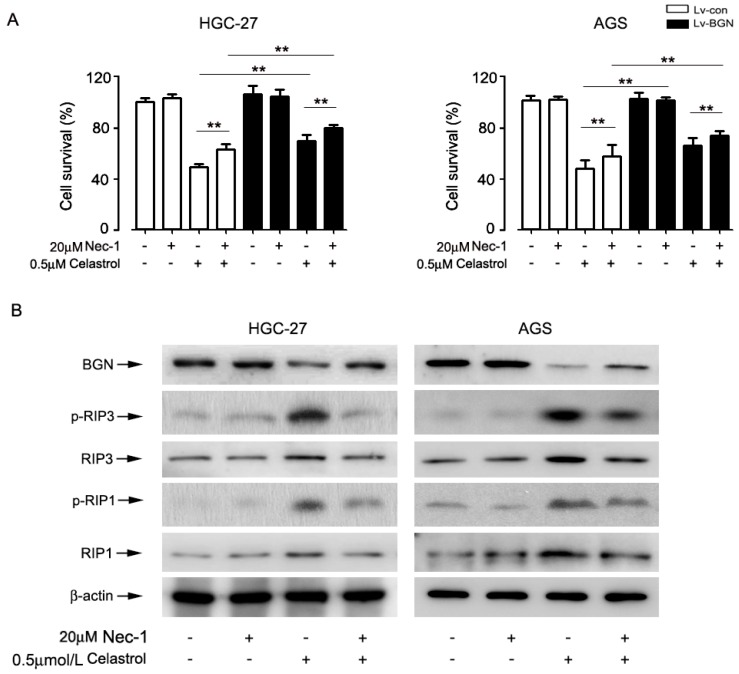
Inhibition of necroptosis by Nec-1 attenuated celastrol-induced cell death in HGC-27 and AGS cells. (**A**) Cells expressing control or over-expressing BGN were pre-treated with necroptosis inhibitor Nec-1 (20 μM) for 2 h and then treated with 0.5 μM celastrol for 24 h. After then, (**A**) the percentage of cell survival was estimated by MTT assay, and (**B**) the expression of p-RIP1, p-RIP3, and BGN was determined by Western blot analysis. Results were presented as the mean ± S.D. of at least three independent experiments. Significant differences compared with controls were indicated as * *P* < 0.05 and ** *P* < 0.01.

To further investigate the relationship between celastrol-mediated BGN expression and necroptosis, cells were incubated with RIP1 inhibitor necrostatin-1 (Nec-1) for 2 h before treatment by celastrol. The MTT result showed that celastrol-induced cell death was significantly blocked by Nec-1; Nec-1 and over-expression BGN had a stronger interference effect on celastrol-induced cell death than alone over-expression BGN or Nec-1 (Figure 5A). In addition, Western blotting showed that Nec-1 markedly inhibited the celastrol-activated p-RIP1and p-RIP3. Interestingly, inhibition necroptosis by Nec-1 up-regulated BGN level in cells exposed to celastrol, signaling a feed-forward regulation of BGN by RIP1 (Figure 5B). The data indicate that celastrol induced cell death in HGC-27 and AGS cells at least in part through BGN-activating necroptosis.

### 2.6. Celastrol Blocking BGN Was Accompanied by Suppressing Pro-Inflammatory Cytokine Production in HGC-27 and AGS Cells

**Figure 6:**
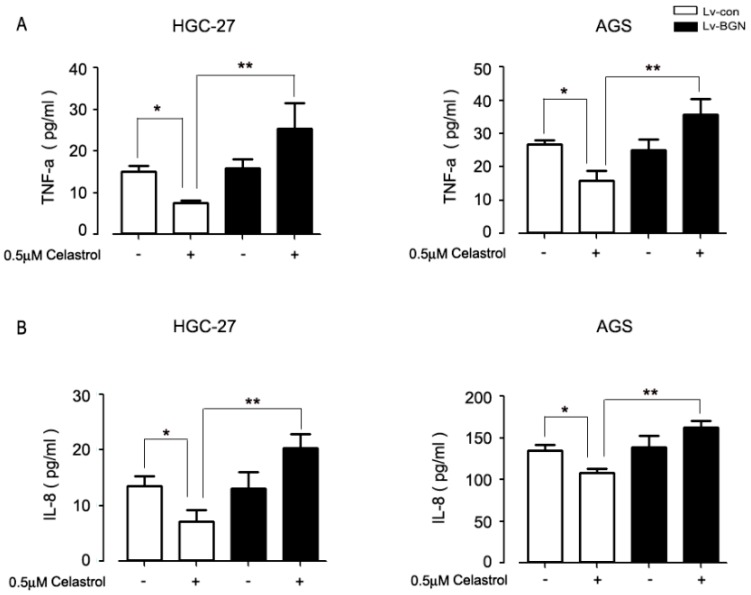
BGN up-regulated celastrol-attenuated inflammatory markers in HGC-27 and AGS cells. Cells expressing control (LV-con) or over-expressing BGN (LV-BGN) were treated with or without 0.5 μM celastrol for 24 h. After then, TNF-α (**A**) and IL-8 (**B**) secretion was determined using ELISA method. Data are the mean ± SD of three independent experiments. Significant differences compared with controls were indicated as * *P* < 0.05 and ** *P* < 0.01.

We then sought to investigate whether celastrol mediated inflammation by targeting BGN in gastric cancer cells; we evaluated the secretion of pro-inflammatory cytokine TNF-α and IL-8. High inflammation always existed in cancer cells. As shown by the data (Figure 6A,B), we found that there was a certain expression of TNF-α and IL-8 in untreated HGC-27 and AGS cells. Celastrol treatment partially inhibited their production in gastric cells and overexpression of BGN restored the level of TNF-α and IL-8 compared to celastrol group. Similar results were shown in HGC-27 and AGS cells. These data suggest that celastrol inhibits the expression of BGN, contributing to attenuate inflammation in gastric cancer cells.

## 3. Discussion

**Figure 7:**
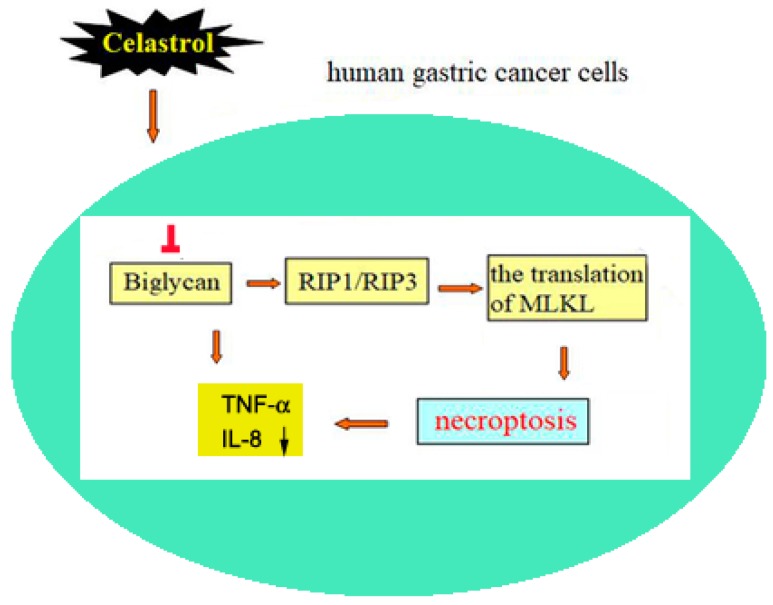
Summary of possibly involved mechanism in anti-tumor effect of celastrol on human gastric cancer cells. Celastrol targeting on inhibiting the expression of BGN significantly promotes the expression of RIP1 and RIP3 and the translation of MLKL from cytoplasm to plasma membrane, leading to the decrease of pro-inflammation cytokines’s production in human HGC-27 and AGScells.

In the present study, we have found a novel role of celastrol as an anti-tumor medicine through reducing the expression of BGN to activate RIP1/RIP3/MLKL signaling pathway, leading to necroptosis and decreased secretion of pro-inflammatory cytokines in HGC-27 and AGS cells (Figure 7). Celastrol-induced necroptosis was confirmed by necrosis-related protein RIP1 and RIP3, and the rescue effects of necroptosis inhibitor Nec-1 and NSA. High expression of BGN was existed in gastric cancer cells, which was down-regulated by celastrol in a dose dependent manner. On the basis of the key role of RIP1/RIP3/MLKL signaling in necroposis [8,40], we proved that celastrol promoted the phosphorylation of RIP1 and RIP3 and plasma membrane translocation of MLKL in HGC-27 and AGS cells, which was prevented by BGN over-expression. Moreover, further results showed that necroptosis inhibitor Nec-1 attenuated the inhibition effect of celastrol on BGN. In addition, celastrol inhibited the production of pro-inflammatory cytokines in HGC-27 and AGS cells by blocking BGN. This study unveils the mechanism of celastrol-induced gastric cancer cell death and underscores the importance of BGN-mediated necroptosis and inflammation in celastrol-induced cell death.

Most patients diagnosed with advanced gastric cancer receive chemotherapeutic treatment [41]. However, the high rate of adverse effects and rapid therapeutic resistance of chemotherapy to limit to treatment is not ginored. Recent studies have shown that chemotherapy normally killed RIP3 and MLKL-deficient tumor cells resulting in apoptosis by activating caspase-3 and yet was unable to reduce their growth in vivo. Interesting, up-regulation of RIP3 alleviated cervical cancer progression and promoted cell’s sensitivity to chemotherapy [42]. Once knockdown of RIP3, the effect of TNF-α strengthening the cytotoxicity of 5-FU in breast cancer cells was abolished [43,44]. Our present study indicated that RIP1/RIP3 was expressed at low levels in two kinds of gastric cancer cells, and celastrol greatly enhanced the expression of RIP1/RIP3 leading to necroptosis, which contributed to gastric cancer cell death. We have no idea whether celastrol promotes the cell’s sensitivity to chemotherapy and radiotherapy against gastric cancer cells. Of course, further studies still are needed to address this issue.

Particularly noteworthy, more and more natural compounds derived from plants or microbes have exhibited promising therapeutic effects for cancer [36,37,38,45]. Celastrol has shown the capacity of inhibiting gastric cancer cells by inducing apoptosis. In our study, surprisingly, we found celastrol induced necroptosis in gastric cancer cells. Our results are in line with the recent Lin’s studies on tanshinone IIA simultaneous induction of apoptosis and necroptosis in human hepatocellular carcinoma HepG2 cells. It is generally believed that necroptosis is incompatible with apoptosis. For example, matrine [36], shikoni [38], and neoalbaconol [45] individually induced necroptosis but not apoptosis in some cancer cell lines. In this study, we noticed that necroptosis inhibitor Nec-1 and NSA in part attenuated celastrol-triggered cell death in HGC-27 and AGS cells, which suggest that expecting apoptosis, celastrol has anticancer ability by inducing necroptosis in gastric cancer cells.

To date, a novel function of biglycan as a signaling molecule and a crucial pro-inflammatory factor has long been appreciated [24,46]. Recently, several research groups have found abnormal expression of BGN in tumors, which is closely related to the migration and poor prognosis of tumors, including gastric cancer [17,18,19,20,21]. The data suggest that higher expression of BGN plays an oncogenic role in cancer migration and invasion [22,23]. Growing evidences have shown that BGN as a danger signal has the pro-inflammatory effect. Mechanistically, it activates some signaling pathway such as Erk, p38, NF-kB by engaging TLR2/4, leading to the synthesis and secretion of pro-inflammatory cytokines and chemokines, such as IL-1, TNF-α. In addition, it was reported that biglycan-deficient mice show improved survival in LPS-induced sepsis and less severe inflammation in both models of sterile inflammatory renal injury and LPS-induced sepsis because of decreased levels of active caspase-1 and mature IL-1 [24,47,48,49]. According to our current knowledge, biglycan-mediated inflammatory milieu may promote tumor growth. On a positive note, for the first time, we found that celastrol inhibited the expression of BGN, and over-expression of BGN prevented celastrol-induced necroptosis in gastric cancer cells. These data indicate that BGN is an important target of celastrol in cancer, and its regulation of necroptosis may be a promising therapeutic strategy for the prevention and treatment of gastric cancer. In regards to the role of proteoglycans in the regulation of necroptosis, it has not been previously documented. Of course, the exact mechanism still needs to be further studied.

A recent study has demonstrated that necroptosis might have anti-inflammatory effects in certain settings, through curbing excessive TNF-α or TLR-induced inflammatory cytokine production. Once the necroptosis effectors, RIPK1 and RIPK3 were blocked, the production of pro-inflammatory cytokine was triggered. In other words, diminished necroptosis can lead to enhanced inflammatory responses [50,51,52]. In our study, we found that celastrol had the ability of anti-inflammatory effects via inhibiting BGN in gastric cancer (Figure 6). Other studies have indicated that celastrol inhibited gastric cancer growth in vivo by induction of apoptosis and autophagy [39]. To further verify the necroptosis of celastrol, we will conduct experiments at the animal level. This will provide a theoretical basis for the promotion of celastrol. Based on above two points, we speculate that the inhibitory effect of celastrol on pro-inflammatory cytokine is attributed to the downregulation of BGN and the activation of necroptosis. These findings support the potential use of celastrol as an effective therapeutic agent for gastric cancer patients.

## 4. Materials and Methods

The primary antibodies against RIP1 (Ser166), RIP1, RIP3, and β-actin were from Cell Signaling Technology (Danvers, MA, USA). Anti-BGN and anti-MLKL antibodies were obtained from Sigma (St Louis, MO, USA). Anti-RIP3 (Ser227) and anti-MLKL (Ser358) antibodies were purchased from Abcam company (Cambridge, MA). Roswell Park Memorial Institute (RPMI)-1640 medium was supplied by Thermo Fisher Scientific, Inc (Logan, UT, USA). Celastrol, Dimethyl sulfoxide (DMSO), cell lysates, 4% paraformaldehyde and 3-(4, 5-dimethylthiazol-2-yl)-2, 5-diphenyltetrazolium bromide (MTT) were purchased from Sigma, whereas 0.05% Trypsin-EDTA and Fetal bovine serum (FBS) were from Invitrogen (Grand Island, NY, USA). Inhibitors Z-VAD-fmk, necrostatin-1 (Nec-1) and necrosulphonamide (NSA) were purchased from TRC (Toronto, Ontario, Canada). Enhanced chemiluminescence solution was purchased from Pierce (Rockford, IL, USA).

### 4.1. Cell Culture

Two human gastric cancer cell lines (human gastric carcinoma HGC-27 cell lines; human gastric adenocarcinoma AGS cell lines) were obtained from American Type Culture Collection (Manassas, VA, USA) and normal gastric epithelial cell lines (GES-1) were purchased from Shanghai cell bank. Cells were grown in RPMI-1640 medium supplemented with 10% FBS, 1% penicillin, and streptomycin. Cells were maintained at 37 °C in a humid incubator (37 °C, 5% CO_2_).

### 4.2. Western Blot Analysis

The cell cultures were harvested and lysed in lysis buffer on ice for 20 min. Determination of protein concentrations were conducted as Bradford’s method. Followed by equivalent amounts of protein boiled for 5 min with sample buffer and cooled on ice, proteins were separated on concentration-appropriate gels by SDS-PAGE and electrophoretically transferred to polyvinylidene diflouride filter (PVDF) membranes (Millipore, Bed ford, MA, USA). After being blocked with 5% non-fat dry milk in TBST for 1 h at room temperature, the membranes were incubated with primary antibodies against β-actin, BGN, RIP1, RIP3, and p-MLKL at 4 °C overnight, subsequently with appropriate secondary antibodies conjugated to horseradish peroxidase (HRP) for 35 min at room temperature. Finally, proteins were detected by an enhanced chemiluminescence solution (Pierce) and analyzed. β-actin was used as a loading control. NIH image J was used to analysis the histogram.

### 4.3. Cell Viability Assay

The AGS and HGC cells were respectively seeded in a flat-bottomed ninety-six-well plate at a density of 1 × 10^5^ cells/mL. The cells were treated with different concentrations of celastrol for 24 h or with/without 0.5 μM celastrol for 24 h following pre-incubation with/without with Nec-1or NSA for 2 h. After incubation, each well was added 10 μL MTT solution (5 mg/mL) for 4 h at 37 °C in a 5% CO_2_ incubator and then added 150 μL DMSO/well to dissolve formazan crystals. The analysis of cell viability was performed using a microplate reader (Molecular Devices, San Jose, CA, USA) at OD 570 nm.

### 4.4. Cell Morphological Analysis

Cells were seeded at a density of 1 × 10^6^ cells/well in a six-well plate. Next day, 0.5 μM celastrol was added. After 24 h, images were taken with an Olympus inverted phase-contrast microscope (Olympus Optical Co., Melville, NY, USA) (200×) equipped with the Quick Imaging system.

### 4.5. Analysis of Cell Death by Flow Cytometry

Cell death was determined using PI exclusion assay. In brief, after treatment, the cells were digested using trypsin without EDTA. Then the cells were repeatedly washed with cold PBS for three times and centrifuged at 1000 rpm for 5 min. Finally, cells were re-suspended and probed by 5 μg/mL PI for 5 min at 4 °C in the dark. Subsequently, the cells undergoing death were immediately analyzed by Guava EasyCyte HT Sampling Flow Cytometer (Merck, Millipore) with an emission wavelength at 630 nm.

### 4.6. Immunofluorescence Assay

The cells were plated in twelve-well plate containing a glass coverslip per well. After 0.5 μM celastrol treatment for 24 h, the cells were washed with PBS three times followed by fixed with 4% paraformaldehyde for 20 min at room temperature. Subsequently, the fixed cells were permeabilized in 0.5% Triton X-100 for 20 min. The cells were blocked with 5% BSA in PBS for 30 min and washed three times with PBS, followed by staining with anti-MLKL antibody (rabbit, 1:100, Abcam, Cambridge, MA, USA) overnight at 4 °C, and then the FITC-labeled anti-rabbit second antibody (1:200, Santa Cruz Biotech, Santa Cruz, CA, USA) incubation for 1 h in the dark was performed. Finally, the cells were probed in anti-fade reagent with DAPI for another 10 min. The cells were rinsed and mounted onto slides and then analyzed and imaged by a Carl Zeiss fluorescent light microscope (Thornwood, NY, USA) using a magnification of 10 × 20 and resolution of 1200 × 1200. Images were acquired with 150 ms exposure times and were taken with up to 20 images per sequence. Red fluorescence represents MLKL protein, and blue fluorescence represents nucleus.

### 4.7. Cytokine Evaluation

The cells were seeded in six-well plate at the density of 1 × 10^5^ per well overnight and then treated with 0.5 μM celastrol for 24 h. Levels of TNF-α and IL-8 in the treated cells were determined using a commercial ELISA kit (KeyGen BioTech, Nanjing, China) according to the manufacturer’s instructions.

### 4.8. Lentivirus Production and Transfection

Lentiviral vector encoding biglycan core protein and green fluorescence protein (GFP) (as control) were purchased from Shanghai Sangon Biotech. For use, monolayer cells grown to about 30% to 50% confluency were infected with lentivirus-containing supernatant for 72 h. Lentiviral vector transfection was performed as described [53].

### 4.9. siRNA Transfection

Human BGN siRNAs, RIP3 siRNAs and non-targeting control siRNA were purchased from Shanghai GenePharma. siRNA transfection was performed as described [53].

### 4.10. Statistical Analysis

All values were represented as mean ± S.D. from triplicate independent experiments per condition. One-way ANOVA test (SPSS 19.0 software, IBM Corporation, Armonk, NY, USA) was applied to analyze the differences between groups. * *P* < 0.05 and ** *P* < 0.01 were accepted to indicate statistically significant differences.

## 5. Conclusions

In conclusion, our study for the first time found that celastrol could decrease the expression of BGN to induce necroptosis and ameliorate inflammation in gastric cancer cells (Figure 7). As a safe clinical drug, celastrol may act as a potential effective drug to treat gastric cancer.

## Abbreviations

BGN	biglycan
FADD	Fas-associated death domain protein
MLKL	mixed-lineage kinase domain-like
Nec-1	necrostatin-1
NSA	necrosulphonamide
SLRP	small leucine repeat proteoglycan family
RIP1 and RIP3	receptor-interacting protein 1 and 3
